# A comprehensive overview of paracetamol poisoning admissions and long-term outcomes in New South Wales, Australia: a retrospective linked data cohort (PAVLOVA-3)

**DOI:** 10.1016/j.lanwpc.2026.101886

**Published:** 2026-05-28

**Authors:** Annabelle S. Chidiac, Nicholas A. Buckley, Firouzeh Noghrehchi, Geoffrey K. Isbister, Angela L. Chiew, Jonathan Brett, Andrew H. Dawson, Darren M. Roberts, Rose Cairns

**Affiliations:** a School of Pharmacy, Faculty of Medicine and Health, The University of Sydney, Sydney, Australia; b New South Wales Poisons Information Centre, The Children's Hospital at Westmead, Sydney, Australia; c Department of Clinical Toxicology, Calvary Mater Newcastle, Waratah, Australia; d School of Medicine and Public Health, University of Newcastle, Newcastle, Australia; e Department of Clinical Toxicology, Prince of Wales Hospital and Community Health Services, Randwick, Australia; f Clinical Pharmacology and Toxicology, St Vincent's Hospital Sydney, Darlinghurst, Australia; g Drug Health, Royal Prince Alfred Hospital, Camperdown, Australia; h Edith Collins Centre, Drug Health Services, Royal Prince Alfred Hospital, Sydney, Australia

**Keywords:** Paracetamol, Acetaminophen, Poisoning, Toxicology, Self-harm, Suicide, Repetition, Survival

## Abstract

**Background:**

Paracetamol poisoning is a major public health issue and a leading cause of acute liver failure. Population-level data on outcomes, repetition, and long-term survival remain limited. This study characterises hospitalised paracetamol poisonings in New South Wales, Australia (population ∼8 million) using a large linked dataset.

**Methods:**

We conducted a retrospective observational study of patients hospitalised with an ICD-10 diagnosis of T39·1 (Poisoning by 4-Aminophenol derivatives), 1st January 2011 to 30th September 2020, using the Poisoning And enVenomation Linkage to evaluate Outcomes and clinical Variation in Australia (PAVLOVA) cohort. Outcomes included intent, demographics, length of stay, Intensive Care Unit admission, liver injury, death, repetition, and long-term post-discharge survival.

**Findings:**

There were 24,092 paracetamol poisoning admissions involving 19,895 individuals. Most admissions were intentional (78·8%, 18,987), and occurred in women (71·0%, 17,095), and the median age was 29 years (IQR, 19–45 years). Intentional poisonings had high rates of psychiatric and substance use comorbidities. Liver injury was more common in accidental poisonings (6·1%, vs intentional poisonings, 1·5%). Intentional poisoning repetition occurred in 21·8% (3374/15,472) of individuals, with greatest risk in the first year. Long-term survival was poorer in those with multiple repetitions: 7·6% (104/1373) had died by study end, compared with 4·7% (571/12,098) of people with no repeated intentional poisoning.

**Interpretation:**

While intentional paracetamol poisonings rarely result in serious liver injury, there is substantial risk of poisoning repetition and long-term mortality. These findings highlight the need for health-system responses focused on post-overdose management and continuity of mental health care.

**Funding:**

NHMRC, Reckitt Benckiser.

**Research in context**

**Evidence before this study**

A previous review by our group used a systematic search of PubMed (between 2017 and 2022), plus Poisons Information Centre annual reports, and direct requests for summary data from global Poisons Information Centres. PubMed search terms included ‘poisoning’ (subheading ‘epidemiology’) OR ‘poisoning’ (subheading ‘mortality’), with no language restrictions. Titles and abstracts were screened for studies that reported paracetamol or analgesic poisoning, and full texts were reviewed to extract information on paracetamol poisonings and outcomes. To capture evidence published since our review, we updated this search on February 6th, 2026, focusing on poisoning repetition and long-term outcomes. Our published review estimated that paracetamol is involved in 6% of poisonings and 56% of severe acute liver injury and acute liver failure. Existing evidence was limited by a reliance on unlinked hospital or poisons centre datasets and substantial data gaps in many countries, including across the Western Pacific Region. Several countries have made regulatory changes to paracetamol availability to reduce harms from poisoning. One population-based study in South Korea examined intentional paracetamol poisonings in adolescents and found increasing rates of repeat poisoning, but was not linked to deaths data and therefore could not assess long-term outcomes. We found no recent population-based studies examining all paracetamol poisoning types, and none assessing paracetamol poisoning repetition and long-term survival.

**Added value of this study**

This study provides a population-based evaluation of paracetamol poisonings in Australia's most populous state, integrating hospital, toxicology, poisons centre and mortality datasets to create a cohort of 19,895 individuals with 24,092 admissions. By capturing all paracetamol poisoning types, rather than selected subgroups, we report the most complete description to date of demographics, clinical characteristics, intent, and outcomes of paracetamol poisoning. Intentional poisonings predominated, occurring mainly in young women with substance use and mental health comorbidities. Intentional poisonings had a lower rate of liver injury than unintentional poisonings (1·5% vs 6·1%). However, one in five individuals had repeated intentional paracetamol poisonings, and the risk is highest in the first year after the index poisoning. Individuals with multiple repeated episodes had markedly poorer long-term survival compared to those who had one event only (7·6% mortality vs 4·7%, respectively).

**Implications of all the available evidence**

Intentional poisonings are the most common type of paracetamol poisoning admission, and while most do not lead to severe liver injury, they carry a substantial risk of repeated self-poisoning and elevated long-term mortality. Regulatory approaches such as pack size restrictions may reduce severity of overdoses but are unlikely to prevent poisoning repetition, which is likely driven by underlying mental health and substance use conditions. Health-system responses need to extend beyond acute medical management to include aftercare and ongoing support following an intentional poisoning event.

## Introduction

Paracetamol is very safe when used appropriately. However, globally, it is estimated to be involved in approximately 6% of poisonings, with paracetamol poisonings increasing in many countries.^1^ The main concern with overdose and poisoning with paracetamol is the risk of acute liver injury, where it is one of the leading causes of acute liver failure in many high-income countries.^1^^,^^2^ Due to the severity of these outcomes, paracetamol has varying degrees of access globally with a number of countries placing restrictions on pack sizes, sales outside of pharmacies, and even enforcing purchasing age restrictions.^1^

In Australia, paracetamol access has been scrutinised due to increasing harms from poisonings.^3^ In 2022 an independent report commissioned by the Therapeutic Goods Administration (TGA, Australia's drug regulator) highlighted increasing poisonings in young people, particularly young women.^4^ In 2023 the TGA announced several changes to paracetamol access, implemented in February 2025 which included pack size restrictions and scheduling changes.^5^ These changes are too recent to be fully evaluated, but similar changes in the United Kingdom, reduced large overdoses, liver unit admissions, transplants, and deaths.^6^

Previous studies on paracetamol overdose have used unlinked datasets, including hospital admissions and poisons centre datasets. These datasets cannot be used to evaluate poisoning repetition: they count events, so one person can appear in the dataset multiple times, but repetition cannot be quantified. In addition, unlinked data cannot be used to study long-term outcomes, including switching to other poisoning/self-harm methods, and deaths.

In this study we aimed to describe patterns and characteristics of hospitalised paracetamol poisonings in New South Wales, Australia (NSW, population ∼8 million) using a large linked statewide data set. Specifically, we aimed to: (i) describe demographics and characteristics of people hospitalised with paracetamol poisoning, (ii) describe treatments and models of care, and (iii) describe outcomes including: length of stay, drug induced liver injury (DILI), deaths, intentional poisoning repetition, and long-term post-discharge survival.

## Methods

### Design and setting

We performed a retrospective observational study of patients admitted to NSW hospitals using the Poisoning And enVenomation Linkage to evaluate Outcomes and clinical Variation in Australia (PAVLOVA) cohort. PAVLOVA is a large data linkage project, described in detail previously.^7^ For this study we used the Admitted Patient Data Collection (APDC) to identify hospitalised paracetamol poisonings, January 2011–September 2020. The APDC contains admissions from all public and private hospitals in NSW, with the exception of Northern Beaches Hospital.^8^ The linked NSW Toxicology dataset (a dataset containing records from the NSW Poisons Information Centre and toxicology units) was used to analyse models of care. The linked Registry of Births, Deaths and Marriages: deaths (referred to as *fact of death* data), was used to evaluate long-term survival, with data available from January 2011–September 2020. The Cause of Death Unit Record File (referred to as *cause of death* data) was used to extract primary and underlying causes of death, available from January 2011–December 2018.

### Cohort selection and classification

APDC diagnoses are coded with the International Classification of Diseases and Related Health Problems, Tenth Revision, Australian Modification (ICD-10-AM, hereafter termed ICD-10). Individuals admitted to hospitals with an ICD-10 diagnosis of T39·1 (*Poisoning by or adverse effect of 4-Aminophenol derivatives*) between January 2011 and September 2020 were included. Cohort entry was defined as the first admission for paracetamol poisoning during the study period. We did not apply a lookback window. Note: the only 4-Aminophenol derivative in therapeutic use is paracetamol. Coingestants were summarised using relevant codes (see Supplementary Methods). We compared demographics and outcomes of paracetamol poisoning admissions with all other poisoning admissions in PAVLOVA (admissions with an ICD-10 diagnosis of T36–T50 (poisoning by drugs, medicaments and biological substances) or T51–T65 (toxic effects of substances chiefly nonmedical as to source), excluding T39·1).

Poisoning intent was classified using ICD-10 external cause codes for: intentional poisonings (X60-X69), accidental poisonings (X40-X49), poisoning of undetermined intent (Y10-Y19), adverse drug reactions (Y40-Y59), and assault (X85-X90). A patient can be assigned up to 51 diagnosis codes in the APDC, and rarely, events could have more than one intent type coded (e.g. an accidental poisoning with one substance and an adverse drug reaction to another). These were treated as follows to ensure mutual exclusivity: any admission with any flag of intentional poisoning was included in the ‘intentional’ group, any other admission with an accidental flag (excluding those with an intentional flag) were included in the ‘accidental’ poisoning group and any admissions which did not have an intentional or accidental flag were then included in the ‘undetermined/other group’ (there were very few adverse drug reaction and assault codes, and so these were combined into ‘other’ to protect the privacy of individuals).

We extracted coded demographics from the APDC. Sex as recorded in the APDC was used for our analyses; it is defined in the APDC data dictionary as ‘the biological sex of the patient’, but in practice it is possible that this field may include sex or gender information. A Charlson Comorbidity Index^9^ was calculated for each individual across all admissions, and additional substance use and psychiatric comorbidities^7^ were extracted.

Outcomes measured included length of stay, DILI (ICD-10 codes K71—Toxic liver disease, and K72—Hepatic failure, not elsewhere classified), dialysis, liver transplant, Intensive Care Unit (ICU) admissions, ventilation, transfers to a psychiatric ward or hospital, transfers to other hospitals, and in-hospital deaths. For a full list of codes, see Supplementary Methods. The APDC does not contain biomarkers or pathology test results, and so we are unable to report on paracetamol serum concentration, liver function tests, or other markers of injury. Our classification of cases of paracetamol poisoning and DILI relies on the presence of the relevant ICD-10 codes. In addition, treatment with N-acetylcysteine (and time to treatment) is not captured in this dataset.

Two models of care were determined, based on whether an individual presented to one of the six NSW hospitals with a dedicated bedside toxicology service, henceforth referred to as a ‘tox hospital’. Those who were transferred from a non tox hospital to a tox hospital (including one with a liver unit) were also analysed as these transfers are likely to reflect higher severity. We also estimated the number of hospital admissions resulting in a call to the NSW Poisons Information Centre, using the linked call data for 2019 (year with most reliable call linkage due to better capture of identifiers).

### Statistical analysis

All statistical analysis was conducted using R (Version 4·3·1). We performed descriptive statistics including median and interquartile range (IQR) for continuous variables, and frequencies and percentages for categorical variables. Missing data is specified in the tables and was not imputed.

Survival analyses were conducted using the *survival* R-package. Survival was defined as the time interval from the date of index event to the date of death. Data were audited for temporal consistency and one record with an implausible date of death (preceding an admission) was identified as a linkage error and right-censored. All other individuals were followed until 30 September 2020 or death, whichever occurred first. Individuals with no death record were right-censored at the end of the follow up period. Results are presented as the cumulative hazard over years. When comparing accidental vs intentional poisonings, the Cox Proportional Hazards Model was used to adjust for age and sex. Results are presented as Hazard Ratios (HRs) with 95% confidence intervals (CIs). The Proportional Hazards assumption was assessed using the Schoenfield residuals and visual inspection of the Kaplan Meier curves. The independence of Schoenfield residuals of time were tested for each covariate individually as well as for the model as a whole using Chi-squared tests.^10^

Results are also presented for stratified age-sex groups, with age grouped into <40 years and ≥40 years. We cut age at 40 because the Proportional Hazards assumption was shown to be valid when stratifying by these pre-specified age-sex groups. Because of low numbers in the undetermined/other group, this group was combined together with the accidental group. A sensitivity analysis was conducted by removing the undetermined/other group.

### Ethics approval

This study was approved by the New South Wales Population and Health Services Human Research Ethics Committee (2019-ETH11677). The requirement for individual consent for access to personal data was waived by the Committee.

### Role of the funding source

The funders had no role in study design, data collection, data analysis, interpretation or writing of the report.

## Results

### Demographics and outcomes of paracetamol poisonings

**Fig. 1:**
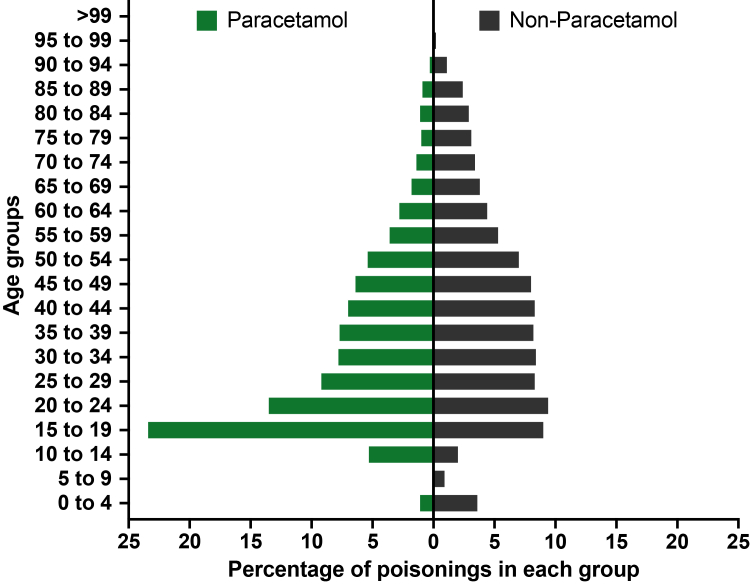
**Age distributions for paracetamol (N = 24,091) vs non-paracetamol poisoning-related admissions (N = 101,350)**.

There were 24,092 paracetamol poisoning admissions (T39·1) to NSW hospitals between January 2011 and September 2020, involving 19,895 individuals. Most admissions were females (71·0%, 17,095) (Table 1). The median duration of follow up between the first recorded paracetamol poisoning admission and the end of available data was 5·1 years (IQR 3·0–7·4 years). Most events (78·8%, 18,987) were intentional (Table 1). The median age was 29 years (IQR 19–45 years) with young patients overrepresented (Fig. 1). Paracetamol poisoning alone (i.e. no pharmaceutical coingestants) occurred in 60·3% (14,536) of events. The median length of stay was 2 days (IQR 2–3 days) and most events were admitted to non tox hospitals (75·5%, 18,190). DILI was reported in 2·4% (589) of admissions and liver transplantation was exceedingly rare (Table 1). The NSW Poisons Information Centre data showed a corresponding call for 32·4% (631/1946) of all admissions in 2019; however, when this was restricted to non tox hospitals this increased to 43·0% (629/1463).

**Table 1:** Demographics and outcomes of paracetamol and non-paracetamol poisoning-related admissions.

	Paracetamol poisoning-related admissions	Non-paracetamol poisoning-related admissions
**Number of admissions**	24,092	101,356
**Number of unique individuals**	19,895	80,069
**External cause**, *n (%)*		
Intentional	18,987 (78·8%)	49,531 (48·9%)
Accidental	4091 (17·0%)	31,765 (31·3%)
Undetermined/other	1014 (4·2%)	20,060 (19·8%)
	n = 24,091	n = 101,350
**Age** (years), *median (IQR)*	28·5 (18·7–44·9)	40·1 (25·0–56·6)
**Sex**, *n (%)*^a^		
Female	17,095 (71·0%)	53,484 (52·8%)
Male	6996 (29·0%)	47,864 (47·2%)
**Country of birth**, *n (%)*		
Australia	19,690 (81·7%)	80,238 (79·2%)
Born outside Australia	4336 (18·0%)	20,559 (20·3%)
Not stated/Inadequately described	66 (0·3%)	559 (0·6%)
**Remoteness**, *n (%)*		
Major cities	15,890 (66·0%)	66,242 (65·4%)
Inner regional	5417 (22·5%)	22,284 (22·0%)
Outer regional	1677 (7·0%)	6874 (6·8%)
Remote	182 (0·8%)	904 (0·9%)
Very remote	100 (0·4%)	532 (0·5%)
Unknown	826 (3·4%)	4520 (4·5%)
**Marital status**, *n (%)*		
Married/defacto	5558 (23·1%)	29,827 (29·4%)
Never married	14,708 (61·0%)	49,358 (48·7%)
Widowed	987 (4·1%)	7526 (7·4%)
Divorced	1419 (5·9%)	7276 (7·2%)
Separated	1189 (4·9%)	5136 (5·1%)
Unknown/blank	222 (0·9%)	2167 (2·1%)
Declined to respond	9 (0·0%)	66 (0·1%)
**Socioeconomic status**, *n (%)*	n = 23,285	n = 97,208
Very low	5512 (23·7%)	23,436 (24·1%)
Low	4565 (19·6%)	19,480 (20·0%)
Middle	4983 (21·4%)	19,850 (20·4%)
High	4023 (17·3%)	17,630 (18·1%)
Very high	4202 (18·0%)	16,812 (17·3%)
	N = 19,895 people	N = 80,069 people
**Charlson score**, *median (IQR)*	0 (0–1)	0 (0–3)
**Model of care**, *n (%)*		
Tox hospital	5902 (24·5%)	24,719 (24·4%)
Non tox hospital	18,190 (75·5%)	76,637 (75·6%)
Transfer to a tox hospital (including liver unit)^b^	279 (1·2%)	726 (0·7%)
**Length of stay** (days), *median (IQR)*^c^	2 (2–3)	2 (2–4)
≤1 day, *n (%)*	2034 (8·4%)	6857 (6·8%)
2 days, *n (%)*	12,246 (50·8%)	55,889 (55·1%)
>2 days, *n (%)*	9812 (40·7%)	38,610 (38·1%)
**Drug induced liver injury**, *n (%)*	589 (2·4%)	458 (0·5%)
**Dialysis**, *n (%)*	147 (0·6%)	760 (0·7%)
**Liver transplant**, *n (%)*	<5	6 (0·0%)
**ICU admission**, *n (%)*	2188 (9·1%)	14,072 (13·9%)
Length of stay (hours), *median (IQR)*^d^	38 (21–66)	34 (18–63)
**Ventilation**, *n (%)*	1025 (4·3%)	7957 (7·9%)
Time spent on ventilation (hours), *median (IQR)*^d^	22 (13–48)	19 (11–41)
**Transfers**	n = 24,090	n = 101,349
To a psychiatric ward/hospital, *n (%)*	7716 (32·0%)	23,740 (23·4%)
To another hospital, *n (%)*	2899 (12·0%)	11,204 (11·1%)
	n = 24,090	n = 101,349
**In-hospital deaths**, *n (%)*	154 (0·6%)	1171 (1·2%)

ICU = Intensive Care Unit; IQR = interquartile range.

a 1 event from the paracetamol group and 8 events from the non-paracetamol group recorded as other/missing observations for sex.

b Individuals in this group come from the non tox hospital group. 7 events from the paracetamol group and 46 events from the non-paracetamol group missing observations for measurements used to determine transfer.

c Does not include stay in a psychiatric unit.

d Reported only for patients in an ICU/ventilated.

Compared to other poisonings, events involving paracetamol were more likely to be intentional and carried out by people who were younger and female. They had higher rates of liver injury (Table 1). Compared to other poisonings, coingestion of paracetamol with opioids and non-steroidal anti-inflammatory drugs (NSAIDs) were more frequent, which are often found in combination products with paracetamol (Supplementary Table S1A and B).

### Characteristics of paracetamol poisonings stratified by intent

**Fig. 2:**
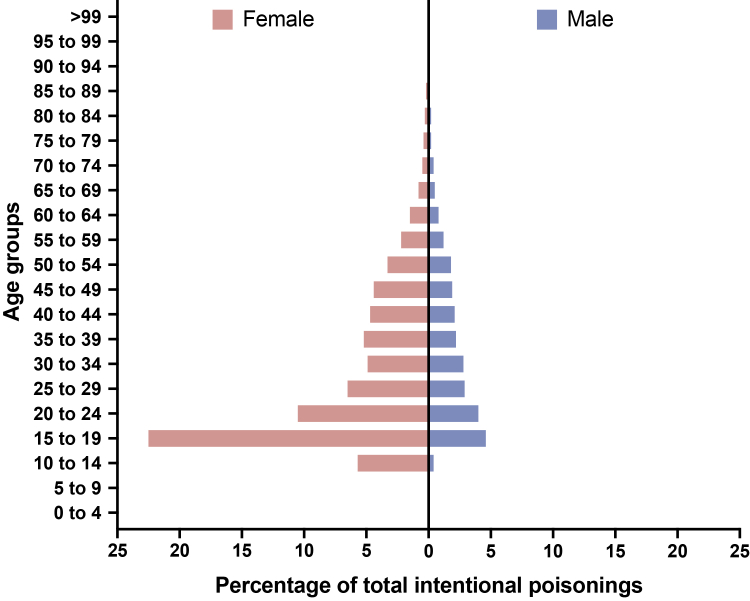
**Age and sex distributions for intentional paracetamol poisoning-related admissions (N = 18,986)**.

We stratified paracetamol poisoning admissions based on coded intent (Table 2). The intentional poisoning group were the youngest, with a median age of 26 years (IQR 18–42 years), and the 15–19 year female age category was the largest subgroup (Fig. 2). Those with an intentional poisoning also had high rates of psychiatric and substance use comorbidities including anxiety (56·5%, 8739/15,472), mood disorders (54·8%, 8479/15,472), and psychoactive substance use (37·5%, 5805/15,472) (Supplementary Table S2). Intentional poisoning events were much more likely to involve a transfer to a psychiatric ward or psychiatric hospital (38·5%, 7303/18,987) (Table 2). DILI occurred in 6·1% (251/4091) of the accidental group and 5·3% (54/1014) of the undetermined/other group, compared to only 1·5% (284/18,987) of the intentional group. The proportion of in-hospital deaths was greatest for the undetermined/other group at 2·0% (20/1014) (Table 2).

**Table 2:** Demographics and outcomes of paracetamol poisoning-related admissions stratified by external cause, N = 24,092.

	Intentional poisoning	Accidental poisoning	Undetermined intent/other
**Number of admissions**	18,987	4091	1014
**Number of unique individuals**	15,472	3944	984
	n = 18,986		
**Age**, *median (IQR)*	26·1 (18·2–41·9)	39·6 (24·0–58·4)	34·7 (22·8–49·0)
**Sex**, *n (%)*	n = 18,986		
Female	14,007 (73·8%)	2445 (59·8%)	643 (63·4%)
Male	4979 (26·2%)	1646 (40·2%)	371 (36·6%)
**Country of birth**, *n (%)*			
Australia	15,718 (82·8%)	3181 (77·8%)	791 (78·0%)
Born outside Australia	3213 (16·9%)	902 (22·0%)	221 (21·8%)
Not stated/Inadequately described	56 (0·3%)	8 (0·2%)	<5
**Remoteness**, *n (%)*			
Major cities	12,341 (65·0%)	2850 (69·7%)	699 (68·9%)
Inner regional	4414 (23·2%)	823 (20·1%)	180 (17·8%)
Outer regional	1354 (7·1%)	252 (6·2%)	71 (7·0%)
Remote	146 (0·8%)	20 (0·5%)	16 (1·6%)
Very remote	75 (0·4%)	20 (0·5%)	5 (0·5%)
Unknown	657 (3·5%)	126 (3·1%)	43 (4·2%)
**Marital status**, *n (%)*			
Married/defacto	4112 (21·7%)	1196 (29·2%)	250 (24·7%)
Never married	12,162 (64·1%)	1970 (48·2%)	576 (56·8%)
Widowed	578 (3·0%)	358 (8·8%)	51 (5·0%)
Divorced	1024 (5·4%)	318 (7·8%)	77 (7·6%)
Separated	935 (4·9%)	210 (5·1%)	44 (4·3%)
Unknown/blank	171 (0·9%)	36 (0·9%)	15 (1·5%)
Declined to respond	5 (0·0%)	<5	<5
**Socioeconomic status**, *n (%)*	n = 18,351	n = 3963	n = 971
Very low	4268 (23·3%)	1015 (25·6%)	229 (23·6%)
Low	3616 (19·7%)	765 (19·3%)	184 (18·9%)
Middle	3950 (21·5%)	811 (20·5%)	222 (22·9%)
High	3177 (17·3%)	669 (16·9%)	177 (18·2%)
Very high	3340 (18·2%)	703 (17·7%)	159 (16·4%)
	N = 15,472 people	N = 3944 people	N = 984 people
**Charlson score**, *median (IQR)*	0 (0–0)	0 (0–3)	0 (0–2)
**Model of care**, *n (%)*			
Tox hospital	4644 (24·5%)	920 (22·5%)	338 (33·3%)
Non tox hospital	14,343 (75·5%)	3171 (77·5%)	676 (66·7%)
Transfer to a tox hospital (including liver unit)^a^	176 (0·9%)	87 (2·1%)	16 (1·6%)
**Length of stay** (days), *median (IQR)*^b^	2 (2–3)	2 (2–5)	2 (2–4)
≤1 day, *n (%)*	1905 (10·0%)	82 (2·0%)	47 (4·6%)
2 days, *n (%)*	9615 (50·6%)	2047 (50·0%)	584 (57·6%)
>2 days, *n (%)*	7467 (39·3%)	1962 (48·0%)	383 (37·8%)
**Drug induced liver injury**, *n (%)*	284 (1·5%)	251 (6·1%)	54 (5·3%)
**Dialysis**, *n (%)*	73 (0·4%)	57 (1·4%)	17 (1·7%)
**Liver transplant**, *n (%)*	<5	0 (0·0%)	<5
**ICU admission**, *n (%)*	1734 (9·1%)	342 (8·4%)	112 (11·0%)
Length of stay (hours), *median (IQR)*^c^	37 (21–62·8)	45·5 (20·3–97·5)	43 (21–110·8)
**Ventilation**, *n (%)*	842 (4·4%)	128 (3·1%)	55 (5·4%)
Time spent on ventilation (hours), *median (IQR)*^c^	21 (13–41)	42 (16–111·5)	33 (15–127·5)
**Transfers**	n = 18,985		
To a psychiatric ward/hospital, *n (%)*	7303 (38·5%)	273 (6·7%)	140 (13·8%)
To another hospital, *n (%)*	2411 (12·7%)	388 (9·5%)	100 (9·9%)
	n = 18,985		
**In-hospital deaths**, *n (%)*	77 (0·4%)	57 (1·4%)	20 (2·0%)

ICU = Intensive Care Unit; IQR = interquartile range.

a Individuals in this group come from the non tox hospital group. 5 events from the intentional group and 2 events from the accidental group missing observations for measurements used to determine transfer.

b Does not include stay in a psychiatric unit.

c Reported only for patients in an ICU/ventilated.

### Models of care

Across all three intent groups, the median length of stay was the same for those presenting to a tox hospital or a non tox hospital (Supplementary Tables S3–S5). However, a greater proportion of admissions in non tox hospitals had a length of stay >2 days (Supplementary Tables S3–S5). Within the accidental group DILI rates were 5·5% (175/3171) for non tox hospitals, vs 8·3% (76/920) for tox hospitals and 29·9% (26/87) for those transferred to a tox hospital. Dialysis, ICU admissions, ventilation, and deaths were greatest for those who had to be transferred to a tox hospital or liver unit (Supplementary Tables S3–S5).

### Repeated intentional poisonings

We divided the ‘intentional’ group according to whether they had no further repeat intentional poisonings, one repeat intentional poisoning or more than one repeat intentional poisoning. Characteristics reported are based on data from the most recent intentional poisoning admission (Table 3). The median duration of follow up for intentional paracetamol admissions was 5·1 years (IQR 3·0–7·5 years). A total of 21·8% (3374/15,472) of people had a repeat intentional poisoning, with 59·3% (2001/15,472) only having one repeat (Table 3). The majority of people who repeat (78·7%, 2655/3374) had their first repeat episode within a year (Supplementary Figure S1A), and 31·2% (1053/3374) had their first repeat within 2 months (Supplementary Figure S1B). Method switching was common, with paracetamol used in 49·4% (988/2001) of initial repeat poisonings, and 39·0% (535/1373) of second and subsequent repeat poisonings (Supplementary Table S1C). The median age was older, and the proportion of females increased, with an increasing number of repeat poisonings (Table 3). Individuals with more than one repeat intentional poisoning had high rates of psychiatric and substance use comorbidities in comparison to those with no further repeats, including anxiety (90·2%, 1239/1373 vs 49·8%, 6020/12,098), mood disorders (89·4%, 1228/1373 vs 47·7%, 5766/12,098), personality and behavioural disorders (79·0%, 1084/1373 vs 19·9%, 2413/12,098), and disorders due to psychoactive substance use (66·9%, 918/1373 vs 32·0%, 3877/12,098) (Supplementary Table S6). People with multiple repeat intentional poisonings were more likely than people with no repeats to be admitted to an ICU (17·6%, 241/1373 vs 8·7%, 1057/12,098 respectively), and to require ventilation (9·5%, 130/1373 vs 4·1%, 502/12,098 respectively). Transfers to a psychiatric ward/hospital were also more common in the group with multiple repeat intentional poisonings (47·9%, 658/1373 vs 34·3%, 4146/12,097 with no repeats, Table 3).

**Table 3:** Demographics and outcomes stratified by intentional poisoning repetition, based on the most recent intentional poisoning recorded in the dataset.

	No Repeats	One repeat poisoning	More than one repeat poisoning	Total
**Number of individuals**	12,098	2001	1373	15,472
**Number using paracetamol (T39**·**1)**, *n (%)*	12,098 (100·0%)	988 (49·4%)	535 (39·0%)	13,621 (88·0%)
	n = 12,097			n = 15,471
**Age**, *median (IQR)*	25·2 (18·0–42·0)	26·2 (18·6–42·1)	31·8 (21·4–45·2)	25·8 (18·3–42·4)
**Sex**, *n (%)*	n = 12,097			n = 15,471
Female	8534 (70·5%)	1494 (74·7%)	1102 (80·3%)	11,130 (71·9%)
Male	3563 (29·5%)	507 (25·3%)	271 (19·7%)	4341 (28·1%)
**Country of birth**, *n (%)*				
Australia	9742 (80·5%)	1707 (85·3%)	1207 (87·9%)	12,656 (81·8%)
Born outside Australia	2311 (19·1%)	287 (14·3%)	162 (11·8%)	2760 (17·8%)
Not stated/Inadequately described	45 (0·4%)	7 (0·3%)	<5	56 (0·4%)
**Remoteness**, *n (%)*				
Major cities	7815 (64·6%)	1270 (63·5%)	938 (68·3%)	10,023 (64·8%)
Inner regional	2784 (23·0%)	504 (25·2%)	303 (22·1%)	3591 (23·2%)
Outer regional	917 (7·6%)	144 (7·2%)	82 (6·0%)	1143 (7·4%)
Remote	108 (0·9%)	12 (0·6%)	5 (0·4%)	125 (0·8%)
Very remote	55 (0·5%)	5 (0·2%)	<5	64 (0·4%)
Unknown	419 (3·5%)	66 (3·3%)	41 (3·0%)	526 (3·4%)
**Marital status**, *n (%)*				
Married/defacto	2932 (24·2%)	400 (20·0%)	231 (16·8%)	3563 (23·0%)
Never married	7526 (62·2%)	1311 (65·5%)	887 (64·6%)	9724 (62·8%)
Widowed	358 (3·0%)	55 (2·7%)	57 (4·2%)	470 (3·0%)
Divorced	613 (5·1%)	120 (6·0%)	107 (7·8%)	840 (5·4%)
Separated	531 (4·4%)	102 (5·1%)	85 (6·2%)	718 (4·6%)
Unknown/blank	135 (1·1%)	10 (0·5%)	6 (0·4%)	151 (1·0%)
Declined to respond	<5	<5	0 (0·0%)	6 (0·0%)
**Socioeconomic status**, *n (%)*	n = 11,734	n = 1945	n = 1336	n = 15,015
Very low	2722 (23·2%)	463 (23·8%)	303 (22·7%)	3488 (23·2%)
Low	2357 (20·1%)	400 (20·6%)	249 (18·6%)	3006 (20·0%)
Middle	2556 (21·8%)	394 (20·3%)	287 (21·5%)	3237 (21·6%)
High	2008 (17·1%)	320 (16·5%)	264 (19·8%)	2592 (17·3%)
Very high	2091 (17·8%)	368 (18·9%)	233 (17·4%)	2692 (17·9%)
**Charlson score**, *median (IQR)*	0 (0–0)	0 (0–1)	0 (0–1)	0 (0–0)
**Model of care**, *n (%)*				
Tox hospital	2876 (23·8%)	481 (24·0%)	381 (27·7%)	3738 (24·2%)
Non tox hospital	9222 (76·2%)	1520 (76·0%)	992 (72·3%)	11,734 (75·8%)
Transfer to a tox hospital (including liver unit)^a^	111 (0·9%)	12 (0·6%)	8 (0·6%)	131 (0·8%)
**Length of stay** (days), *median (IQR)*^b^	2 (2–3)	2 (2–3)	2 (2–3)	2 (2–3)
≤1 day, *n (%)*	1109 (9·2%)	263 (13·1%)	179 (13·0%)	1551 (10·0%)
2 days, *n (%)*	6329 (52·3%)	984 (49·2%)	661 (48·1%)	7974 (51·5%)
>2 days, *n (%)*	4660 (38·5%)	754 (37·7%)	533 (38·8%)	5947 (38·4%)
**Drug induced liver injury**, *n (%)*	189 (1·6%)	28 (1·4%)	15 (1·1%)	232 (1·5%)
**Dialysis**, *n (%)*	53 (0·4%)	6 (0·3%)	<5	63 (0·4%)
**Liver transplant**, *n (%)*	<5	0 (0·0%)	0 (0·0%)	<5
**ICU admission**, *n (%)*	1057 (8·7%)	282 (14·1%)	241 (17·6%)	1580 (10·2%)
Length of stay (hours), *median (IQR)*^c^	37 (21–63)	40 (21–63·8)	38 (21–60)	38 (21–63)
**Ventilation**, *n (%)*	502 (4·1%)	143 (7·1%)	130 (9·5%)	775 (5·0%)
Time spent on ventilation (hours), *median (IQR)*^c^	21 (13–43)	22 (14·5–43)	20·5 (12–38)	21 (13–42)
**Transfers**	n = 12,097			n = 15,471
To a psychiatric ward/hospital, *n (%)*	4146 (34·3%)	880 (44·0%)	658 (47·9%)	5684 (36·7%)
To another hospital, *n (%)*	1444 (11·9%)	241 (12·0%)	146 (10·6%)	1831 (11·8%)
	n = 12,097			n = 15,471
**In-hospital deaths**, *n (%)*	69 (0·6%)	5 (0·2%)	7 (0·5%)	81 (0·5%)

ICU = Intensive Care Unit; IQR = interquartile range.

a Individuals in this group come from the non tox hospital group. 6 events in total missing observations for measurements used to determine transfer, 3 from the no repeats group, 2 from the one repeat group and 1 from the more than one repeat group.

b Does not include stay in a psychiatric unit.

c Reported only for patients in an ICU/ventilated.

### Long-term outcomes and survival

A total of 1382/19,895 (6·9%) deaths were recorded by the end of the study period with a high incidence of injury deaths, including from paracetamol poisoning (Supplementary Table S7A). The accidental poisoning group had the greatest proportion of deaths (13·8% deceased, 543/3944) (Supplementary Table S7B). When adjusted for age and sex, the accidental/undetermined group had a greater risk of death compared to the intentional group, HR 1·53 (95% CI 1·36, 1·70) (Supplementary Table S8).

**Fig. 3:**
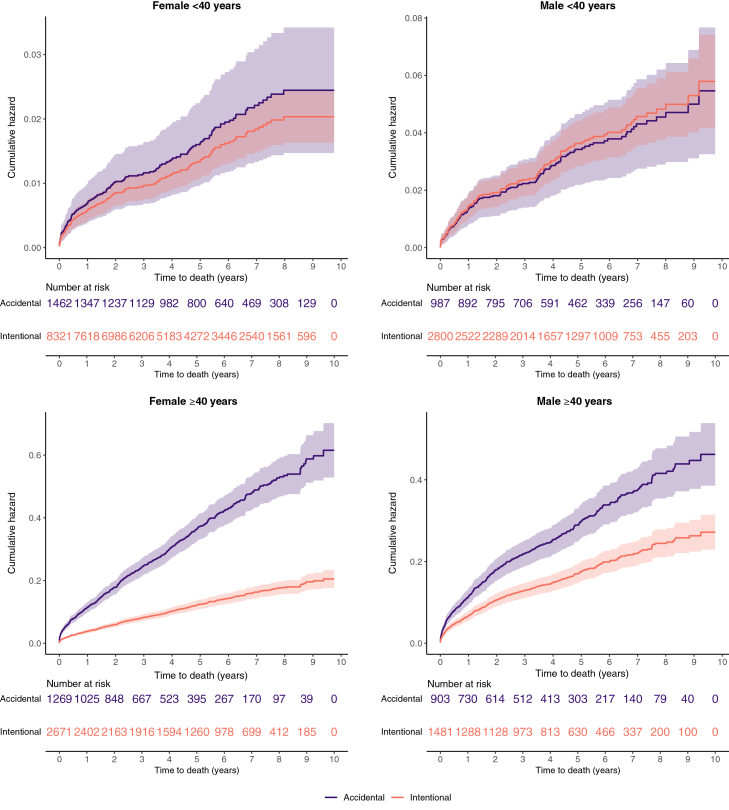
**Survival curves comparing accidental (purple) vs intentional (pink) poisoning groups (including 95% confidence intervals) for age-sex subgroups (N = 19,894).** Note: Accidental and undetermined/other poisoning events have been combined due to low numbers. Purple and pink shaded areas represent confidence intervals. Results are stratified by age and sex in this figure. In the results text we present an age- and sex-adjusted model. One individual was not included in this figure as their age was missing.

In addition to the age- and sex-adjusted model, long-term survival by poisoning intent type was presented stratified by age (cut at 40 years), and sex. The greatest difference was observed for individuals ≥40 years (Fig. 3). The top five causes of death for the intentional poisoning group were mostly injuries (hanging, asphyxiation, accidental, and intentional poisonings), while for the accidental poisoning group they were mostly natural causes (including cardiovascular and respiratory diseases, and sepsis, Supplementary Table S7B).

**Fig. 4:**
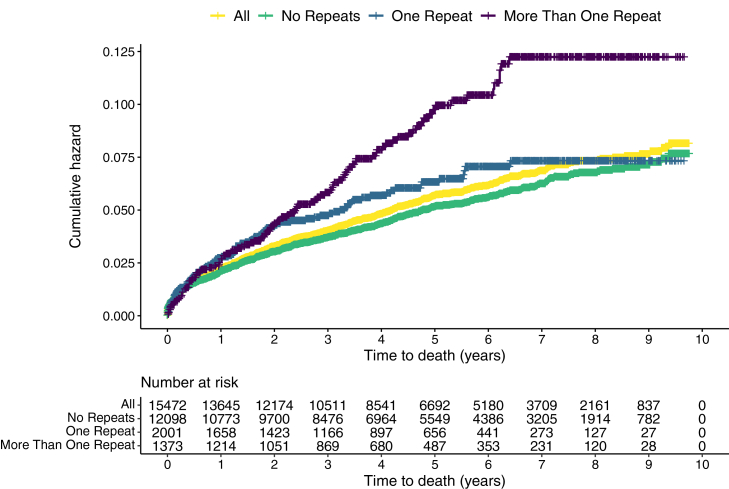
**Survival curves for people with an intentional paracetamol poisoning-related admission stratified by number of intentional poisoning repetitions (N = 15,472).** Note: Time 0 starts at the second event for those with one repeat intentional poisoning and the third event for those with more than one repeat intentional poisoning.

We also examined long-term survival stratified by intentional poisoning repetition. At the end of the study period those with more than one repeat had the greatest proportion of deaths recorded (7·6%, 104/1373) (Supplementary Table S7C) and the lowest survival (Fig. 4). Top causes of death were similar across all three groups (Supplementary Table S7C).

## Discussion

This study highlights the ongoing burden of paracetamol poisonings in New South Wales—the most populous state in Australia. Most paracetamol poisonings were intentional, with most occurring in young women. Individuals with intentional paracetamol poisonings had high rates of psychiatric and substance use comorbidities, consistent with intentional self-harm generally.^11^ These comorbidities increased with an increasing number of repeat intentional poisonings. Rates of liver injury were greater amongst accidental and undetermined/other paracetamol poisonings. Long-term survival was also lowest amongst accidental poisonings. Over one fifth of people who had an intentional paracetamol poisoning went on to have another poisoning, and those with multiple repeat intentional poisonings also had poorer long-term survival.

Our findings regarding the burden of intentional paracetamol poisoning in young women are consistent with the literature.^3^^,^^4^^,^^12^, ^13^, ^14^, ^15^, ^16^, ^17^ Intentional poisonings tend to be impulsive events, with people consuming tablets on hand at the time of acute crisis.^4^ Many countries have reduced the accessibility of paracetamol, aimed at reducing risk of harm from impulsive overdoses. Denmark's imposed age restriction, limiting the purchase of non-opioid analgesics to those aged 18 and over, was associated with a 17% reduction in poisoning admissions in people aged <18 years.^18^ Various European countries have restrictions on paracetamol availability, with many not allowing non-pharmacy sales, and restricting pack sizes.^19^ The United Kingdom introduced pack size restrictions in 1998, which saw a decrease in paracetamol overdoses, large overdoses, liver unit admissions, and mortality.^20^, ^21^, ^22^ Similar to the United Kingdom, Australia has recently restricted pack sizes.^5^ However, this change is relatively new and not captured in the dataset used in the current study. Conversely, some countries have broadened paracetamol access. In South Korea, legislation to allow sales of paracetamol in convenience stores and supermarkets was introduced in 2012, and was associated with increasing intentional poisonings with paracetamol among adolescents, and increasing repeat self-poisoning.^23^

Accidental poisonings had higher rates of liver injury and poorer long-term survival. Although lower doses are taken in these events when compared to intentional poisonings,^13^^,^^24^ the symptoms of liver injury are not immediate. Individuals with accidental poisonings often do not realise they have taken a dangerous dose, which can result in delayed presentation, by which time hepatotoxicity has already occurred.^13^^,^^25^ While it is not possible to understand the drivers of these accidental poisonings from the admitted patient dataset, other studies using poisons information centre data have indicated multiple factors at play. This includes inadequate pain control, and simultaneous use of multiple paracetamol-containing products.^26^ In addition, errors can occur due to lack of understanding of the risks of toxicity and the lack of knowledge of the correct dose regimen.^27^^,^^28^ Evidence for the efficacy of paracetamol is lacking for many common pain conditions.^29^ Improving referral pathways to chronic pain service could address underlying drivers of dose escalation. In addition, pharmacist led medication reviews are a potential strategy to address inappropriate polypharmacy and optimise pain management.^30^ These could be targeted to people hospitalised with accidental poisoning to prevent future events and drug-related harms. In addition, public education about appropriate dosing and not combining different paracetamol-containing products is a possible strategy, however Australia already has mandatory high-visibility warning statements on paracetamol products, and these have not been shown to be effective in a simulated patient study.^27^ In addition, in Canada, updated labelling standards found no significant impact on hospital admission rates for accidental paracetamol overdose.^31^ The recent Australian paracetamol pack size restrictions were not aimed at reducing harm from accidental overdose. However, they could have an impact through awareness raising from the heavily publicised re-scheduling, and by reducing overall quantities of paracetamol available in the home.

Most individuals presented to hospitals without a dedicated bedside toxicology service. This is expected. New South Wales had only six hospitals with a bedside toxicology service at the time of the study. A greater proportion of admissions presenting to tertiary toxicology hospitals had a length of stay ≤2 days when compared to cases admitted to hospitals without a dedicated bedside toxicology service. This potentially represents a significant cost saving for the health care system. Over 40% of the admissions from hospitals without a dedicated bedside toxicology service resulted in a call to the New South Wales Poisons Information Centre. Despite there being national guidelines on the treatment of paracetamol poisoning,^32^ paracetamol overdose can be complex and require specialist input. This highlights the importance of poisons centres to guide clinical care, particularly for hospitals without a toxicology service.

Individuals with more than one repeat intentional poisoning were found to have the worst survival. Compared to individuals with only one intentional poisoning, those with more than one repeat intentional poisoning had approximately twice the prevalence of anxiety, mood and psychoactive substance use disorders, and approximately four times the prevalence of personality and behavioural disorders. The high rates of psychiatric and substance use comorbidities in this population of repeat presenters make them a high-risk group for repeated self-harm^33^ and eventual suicide.^34^ Over 20% had a repeat intentional poisoning, with >75% of initial repeats occurring within the first year. This is similar to a systematic review which quoted an overall one-year self-harm repetition rate of 18%.^35^ Although deaths were infrequent, with high rates of repetition, intervention and follow up within the first year is vital. A postcard intervention was found to reduce the number of repeat episodes per person.^36^ Psychological or psychosocial interventions, such as cognitive behavioural therapy and dialectical behaviour therapy, have the potential to reduce repeated episodes of self-harm and the proportion of individuals repeating self-harm however they are supported with only low to moderate quality evidence.^37^^,^^38^

While the group who had repeated self-poisonings were particularly at risk for worse long-term outcomes, another study with the PAVLOVA dataset has highlighted the elevated risk of premature death following any self-poisoning admission.^39^ This highlights the need for aftercare following all self-poisoning admissions, and viewing these events as opportunities for intervention to prevent future poisonings and deaths. There is considerable unexplained variability in the effectiveness of aftercare services, however, they are likely to result in net cost savings for the health system.^40^ Linked data studies are essential for monitoring the uptake and true effectiveness of such programs.

### Limitations

The Admitted Patient Data Collection underestimates the true frequency of paracetamol poisonings, as it does not capture cases not admitted, e.g. those treated in the emergency department or those who can be safely managed at home (approximately 75% of dosing errors^26^). Using the ICD-10 codes we classified individuals according to their intent however in the hospital setting this is often hard to interpret and exposures may have been misclassified. We classified the first intentional poisoning event in the dataset as the ‘index event’, however we are unable to know whether these individuals had events prior to 2010. Additionally, data were only available until 2020 at the time of this study. Future studies could use an updated dataset to examine patterns of paracetamol poisoning in response to recent events including the Coronavirus disease 2019 (COVID-19) pandemic and paracetamol pack size changes in Australia. The admitted patient data does not contain information regarding the dose taken, formulation used or pack size, which would allow for better identification of high-risk products. It also does not capture other estimates of poisoning severity such as a poisoning severity score or serum paracetamol concentrations. Treatment with N-acetylcysteine is not recorded in the admitted patient data, and some treatments such as dialysis and ventilation may be required for coingestants rather than paracetamol. The admitted patient data does not contain linked laboratory results, meaning liver function test results are not available. Diagnosis of liver injury in the admitted patient data requires a liver disease diagnosis on the discharge summary, and it is likely that many people had small rises in liver function tests indicating hepatotoxicity, without receiving a liver injury diagnosis code. As such, rates of liver injury may be underreported.

Intrinsic limitations of the population-based linked data design include the geographic restriction to New South Wales. We were unable to account for interstate or international migration, which may result in unmeasured loss to follow-up. In addition, all data linkage carries risk of false linkage and missed linkage. This occurred with one individual in our study however, probabilistic methods used by the NSW Centre for Health Records Linkage (CheReL) data linkage unit are robust and result in an acceptably low linkage error rate. Additional data cleaning and validation steps were performed prior to analysis, as described in the PAVLOVA cohort profile.^7^

### Conclusions

Paracetamol is a common cause of poisoning admitted to Australian hospitals. Intentional poisonings constitute most events which supports government decisions to reduce paracetamol pack sizes to limit doses taken in impulsive overdoses. Our data linkage identified high rates of repeat intentional poisoning, with repetition associated with poorer long-term survival. Pack size restrictions are unlikely to impact poisoning repetition, highlighting the importance of secondary prevention through aftercare in people who have intentional poisonings. In addition, we found that accidental poisonings had higher rates of liver injury and death. Recent government interventions may reduce the likelihood of large accidental overdoses. Other measures should be considered including education regarding acute pain management options and safe dosing of paracetamol.

## Contributors

RC and NAB were responsible for the conceptualisation and methodology of the study. RC obtained funding. ASC and FN conducted the formal analysis and visualisation. JB, GKI, AHD, DMR and ALC contributed to the resources of the study. ASC wrote the original draft and all authors contributed to the review and editing of subsequent drafts. ASC and FN accessed and verified the data. All authors read and approved the final version.

## Data sharing statement

The PAVLOVA dataset is only accessible by named investigators on the ethics approval. Requests for linked APDC and deaths data can be made to the NSW Centre for Health Records Linkage (CHeReL).

## Declaration of interests

ASC is supported by a PhD scholarship funded by Reckitt Benckiser, as part of an untied educational grant awarded to RC. RC has also received conference speaker fees/honoraria from Reckitt Benckiser and The Pharmacy Guild of Australia. These funders had no role in the design, conduct, or interpretation of the study's findings. All other authors have no conflicts of interest to be declared.
