# Supplementary material for: A comprehensive overview of paracetamol poisoning admissions and long-term outcomes in New South Wales, Australia: a retrospective linked data cohort (PAVLOVA-3)
Source: Lancet Reg Health West Pac. 2026 May 28;71:101886. doi: 10.1016/j.lanwpc.2026.101886 (PMC13233768; doi:10.1016/j.lanwpc.2026.101886)
Supplement: Supplementary Figure and Tables [file mmc1.docx]

**Supplementary Material for ‘A comprehensive overview of paracetamol poisoning admissions and long-term outcomes in New South Wales, Australia: a retrospective linked data cohort (PAVLOVA-3)’**

**Table of Contents**

[Supplementary Methods 2](#_Toc228455326)

[Supplementary Table 1A 3](#_Toc228455327)

[Supplementary Table 1B 3](#_Toc228455328)

[Supplementary Table 1C 3](#_Toc228455329)

[Supplementary Table 2 4](#_Toc228455330)

[Supplementary Table 3 5](#_Toc228455331)

[Supplementary Table 4 6](#_Toc228455332)

[Supplementary Table 5 7](#_Toc228455333)

[Supplementary Table 6 8](#_Toc228455334)

[Supplementary Table 7A 9](#_Toc228455335)

[Supplementary Table 7B 9](#_Toc228455336)

[Supplementary Table 7C 10](#_Toc228455337)

[Supplementary Table 8 11](#_Toc228455338)

[Supplementary Figure 1A 12](#_Toc228455339)

[Supplementary Figure 1B 13](#_Toc228455340)

[STROBE Statement 14](#_Toc228455341)

# Supplementary Methods

| **Demographic/outcome** | **Measurement used** |
| --- | --- |
| External cause code^*^ | diagnosis_codeP, diagnosis_code1- diagnosis_code50   - Intentional - X60-X69 - Accidental - X40-X49 - Undetermined - Y10-Y19 - Adverse Drug Reactions - Y40-Y59 - Assault - X85-X90 |
| Age | age_recode |
| Sex | sex_group |
| Country of birth | country_of_birth_group |
| Remoteness | remoteness_group |
| Marital status | marital_group |
| Socioeconomic status | sa2_2011_code  Ranking within State or Territory Deciles   - Very low = 1,2 - Low = 3,4 - Middle = 5,6 - High = 7,8 - Very High = 9,10   (Obtainable from *2033.0.55.001 - Census of Population and Housing: Socio-Economic Indexes for Areas (SEIFA), Australia, 2011 -* Statistical Area Level 2, Indexes, SEIFA 2011 Table 2) |
| Charlson score, Charlson groupings and other comorbidities not captured by Charlson^*^ | age_recode  diagnosis_codeP, diagnosis_code1- diagnosis_code50 |
| Coingestants^*^ | diagnosis_codeP, diagnosis_code1- diagnosis_code50 |
| Model of care | facility_identifier_recode  mode_of_separation_recode  facility_trans_to_recode |
| Length of stay | episode_length_of_stay  days_in_psych_unit  Length of stay inclusive of episode start and end dates |
| Drug induced liver injury^*^ | diagnosis_codeP, diagnosis_code1- diagnosis_code50   - K71 (Toxic liver disease), K72 (Hepatic failure, not elsewhere classified) |
| Dialysis | procedure_codeP, procedure_code1- procedure_code50   - 13100-00, 13100-01, 13100-02, 13100-03, 13100-04, 13100-05, 13100-06, 13100-07, 13100-08, 13750-06, 90225-02 |
| Liver transplant | procedure_codeP, procedure_code1- procedure_code50   - 90317-00 |
| ICU admission | hours_in_icu |
| Ventilation | hours_on_mech_ventilation |
| Transfer to psychiatric ward/hospital | mode_of_separation_recode   - 4   days_in_psych_unit |
| Transfer to another hospital | mode_of_separation_recode   - 5 |
| In-hospital deaths | mode_of_separation_recode   - 6, 7 |
| Deaths by end of study period | death_date (from Register of Births, Deaths and Marriages) |
| Cause of death | RACS0, RACS1, RACS2, RACS3, RACS4, RACS5, RACS6, RACS7, RACS8, RACS9, RACS10, RACS11, RACS12 (from the Cause of Death Unit Record File) |

ICU = Intensive Care Unit.

^*^Diagnosis codes are classified according to the International Classification of Diseases and Related Health Problems, Tenth Revision, Australian Modification (ICD-10-AM). These are input by individuals with specialist training based on the patient’s healthcare record.

Supplementary Table 1A**:** Top 5 coingestants for paracetamol and non-paracetamol poisoning-related admissions, *n (%)*.

| **Paracetamol Poisoning-Related Admissions** | | **Non-Paracetamol Poisoning-Related Admissions** | |
| --- | --- | --- | --- |
| **Number of admissions** | 24092 | **Number of admissions** | 101356 |
| **Coingestants** |  | **Coingestants** |  |
| **T40.2** Other opioids | 7199 (29·9%) | **T42.4** Benzodiazepines | 21696 (21·4%) |
| **T39.3** Other NSAIDs | 3663 (15·2%) | **T43.5** Other and unspecified antipsychotics and neuroleptics | 13631 (13·4%) |
| **T51.0** Ethanol | 3347 (13·9%) | **T43.2** Other and unspecified antidepressants | 12244 (12·1%) |
| **T42.4** Benzodiazepines | 3286 (13·6%) | **T51.0** Ethanol | 11770 (11·6%) |
| **T43.2** Other and unspecified antidepressants | 3053 (12·7%) | **T40.2** Other opioids | 8343 (8·2%) |

NSAID = non-steroidal anti-inflammatory drug.

Supplementary Table 1B**:** Top 5 coingestants for paracetamol poisoning-related admissions stratified by external cause, *n (%)*.

| **Intentional Poisoning** | | **Accidental Poisoning** | | **Undetermined Intent/Other** | |
| --- | --- | --- | --- | --- | --- |
| **Number of admissions** | 18987 | **Number of admissions** | 4091 | **Number of admissions** | 1014 |
| **Coingestants** |  | **Coingestants** |  | **Coingestants** |  |
| **T40.2** Other opioids | 5823 (30·7%) | **T40.2** Other opioids | 1039 (25·4%) | **T40.2** Other opioids | 337 (33·2%) |
| **T39.3** Other NSAIDs | 3220 (17·0%) | **T39.3** Other NSAIDs | 324 (7·9%) | **T39.3** Other NSAIDs | 119 (11·7%) |
| **T51.0** Ethanol | 3022 (15·9%) | **T42.4** Benzodiazepines | 316 (7·7%) | **T51.0** Ethanol | 112 (11·0%) |
| **T42.4** Benzodiazepines | 2859 (15·1%) | **T45.0** Antiallergic and antiemetic drugs | 230 (5·6%) | **T42.4** Benzodiazepines | 111 (10·9%) |
| **T43.2** Other and unspecified antidepressants | 2844 (15·0%) | **T51.0** Ethanol | 213 (5·2%) | **T45.0** Antiallergic and antiemetic drugs | 100 (9·9%) |

NSAID = non-steroidal anti-inflammatory drug.

Supplementary Table 1C**:** Top 5 coingestants for people who have had intentional paracetamol poisoning-related admissions stratified by intentional poisoning repetition, based on the most recent intentional poisoning recorded in the dataset, *n (%)*.

| **No Repeats** | | **One Repeat Poisoning** | | **More Than One Repeat Poisoning** | |
| --- | --- | --- | --- | --- | --- |
| **Number of admissions** | 12098 | **Number of admissions** | 2001 | **Number of admissions** | 1373 |
| **T39.1 4-Aminophenol derivatives** | 12098 (100·0%) | **T39.1 4-Aminophenol derivatives** | 988 (49·4%) | **T39.1 4-Aminophenol derivatives** | 535 (39·0%) |
| **Coingestants** |  | **Coingestants** |  | **Coingestants** |  |
| **T40.2** Other opioids | 3789 (31·3%) | **T43.2** Other and unspecified antidepressants | 434 (21·7%) | **T43.5** Other and unspecified antipsychotics and neuroleptics | 407 (29·6%) |
| **T39.3** Other NSAIDs | 2249 (18·6%) | **T42.4** Benzodiazepines | 389 (19·4%) | **T42.4** Benzodiazepines | 303 (22·1%) |
| **T51.0** Ethanol | 2023 (16·7%) | **T43.5** Other and unspecified antipsychotics and neuroleptics | 388 (19·4%) | **T43.2** Other and unspecified antidepressants | 250 (18·2%) |
| **T43.2** Other and unspecified antidepressants | 1763 (14·6%) | **T40.2** Other opioids | 321 (16·0%) | **T51.0** Ethanol | 247 (18·0%) |
| **T42.4** Benzodiazepines | 1738 (14·4%) | **T51.0** Ethanol | 319 (15·9%) | **T40.2** Other opioids | 185 (13·5%) |

NSAID = non-steroidal anti-inflammatory drug**.**

Supplementary Table 2**:** Charlson groupings for people with paracetamol poisoning-related admissions stratified by external cause.

|  | **Intentional Poisoning** | **Accidental Poisoning** | **Undetermined Intent/Other** | **All Poisonings** |
| --- | --- | --- | --- | --- |
| **Number of individuals** | 15472 | 3944 | 984 | 19895 |
| **Charlson groupings**, *n (%)* |  |  |  |  |
| Myocardial infarction | 236 (1·5%) | 162 (4·1%) | 24 (2·4%) | 407 (2·0%) |
| Congestive heart failure | 212 (1·4%) | 294 (7·5%) | 52 (5·3%) | 529 (2·7%) |
| Peripheral vascular disease | 93 (0·6%) | 75 (1·9%) | 18 (1·8%) | 177 (0·9%) |
| Cerebrovascular disease | 297 (1·9%) | 188 (4·8%) | 42 (4·3%) | 506 (2·5%) |
| Dementia | 91 (0·6%) | 151 (3·8%) | 14 (1·4%) | 249 (1·3%) |
| Chronic pulmonary disease | 898 (5·8%) | 427 (10·8%) | 105 (10·7%) | 1339 (6·7%) |
| Rheumatic disease | 69 (0·4%) | 44 (1·1%) | 10 (1·0%) | 119 (0·6%) |
| Peptic ulcer disease | 207 (1·3%) | 140 (3·5%) | 39 (4·0%) | 351 (1·8%) |
| Mild liver disease | 144 (0·9%) | 125 (3·2%) | 31 (3·2%) | 276 (1·4%) |
| Diabetes without chronic complication | 924 (6·0%) | 433 (11·0%) | 98 (10·0%) | 1395 (7·0%) |
| Diabetes with chronic complication | 702 (4·5%) | 526 (13·3%) | 124 (12·6%) | 1302 (6·5%) |
| Hemiplegia or paraplegia | 376 (2·4%) | 220 (5·6%) | 56 (5·7%) | 612 (3·1%) |
| Kidney disease | 488 (3·2%) | 498 (12·6%) | 80 (8·1%) | 1038 (5·2%) |
| Any malignancy, including lymphoma and leukaemia, except malignant neoplasm of the skin | 674 (4·4%) | 442 (11·2%) | 62 (6·3%) | 1146 (5·8%) |
| Moderate or severe liver disease | 405 (2·6%) | 357 (9·1%) | 96 (9·8%) | 783 (3·9%) |
| Metastatic solid tumour | 786 (5·1%) | 564 (14·3%) | 114 (11·6%) | 1398 (7·0%) |
| AIDS/HIV | 144 (0·9%) | 24 (0·6%) | 12 (1·2%) | 168 (0·8%) |
| **Other comorbidities not captured by Charlson**, *n (%)* |  |  |  |  |
| Mental disorders due to known physiological conditions (F01-F09) | 937 (6·1%) | 608 (15·4%) | 115 (11·7%) | 1567 (7·9%) |
| Mental and behavioural disorders due to psychoactive substance use (F10-F19) | 5805 (37·5%) | 1368 (34·7%) | 450 (45·7%) | 7282 (36·6%) |
| Schizophrenia, schizotypal, delusional, and other non-mood psychotic disorders (F20-F29) | 1168 (7·5%) | 264 (6·7%) | 103 (10·5%) | 1437 (7·2%) |
| Mood [affective] disorders (F30-F39) | 8479 (54·8%) | 947 (24·0%) | 381 (38·7%) | 9422 (47·4%) |
| Anxiety, dissociative, stress-related, somatoform and other nonpsychotic mental disorders (F40-F48) | 8739 (56·5%) | 1138 (28·9%) | 434 (44·1%) | 9903 (49·8%) |
| Behavioural syndromes associated with physiological disturbances and physical factors (F50-F59) | 1025 (6·6%) | 157 (4·0%) | 62 (6·3%) | 1162 (5·8%) |
| Disorders of adult personality and behaviour (F60-F69) | 4414 (28·5%) | 476 (12·1%) | 217 (22·1%) | 4815 (24·2%) |
| Intellectual disabilities (F70-F79) | 247 (1·6%) | 63 (1·6%) | 26 (2·6%) | 297 (1·5%) |
| Pervasive and specific developmental disorders (F80-F89) | 323 (2·1%) | 46 (1·2%) | 19 (1·9%) | 356 (1·8%) |
| Behavioural and emotional disorders with onset usually occurring in childhood and adolescence (F90-F98) | 945 (6·1%) | 128 (3·2%) | 44 (4·5%) | 1059 (5·3%) |
| Unspecified mental disorder (F99) | 160 (1·0%) | 26 (0·7%) | 10 (1·0%) | 182 (0·9%) |

AIDS/HIV = acquired immunodeficiency syndrome/human immunodeficiency virus.

Supplementary Table 3**:** Model of care vs outcomes for intentional paracetamol poisoning-related admissions.

|  | **Tox Hospital** | **Non Tox Hospital** | **Transfer to Tox Hospital (including liver unit)*** |
| --- | --- | --- | --- |
| **Number of admissions** | 4644 | 14343 | 176 |
| **Length of stay** (days), *median (IQR)*^Δ^ | 2 (2-3) | 2 (2-3) | 4 (2-9) |
| ≤1 day, *n (%)* | 332 (7·1%) | 1573 (11·0%) | 10 (5·7%) |
| 2 days, *n (%)* | 2961 (63·8%) | 6654 (46·4%) | 35 (19·9%) |
| >2 days, *n (%)* | 1351 (29·1%) | 6116 (42·6%) | 131 (74·4%) |
| **Drug induced liver injury**, *n (%)* | 88 (1·9%) | 196 (1·4%) | 26 (14·8%) |
| **Dialysis**, *n (%)* | 33 (0·7%) | 40 (0·3%) | <5 |
| **Liver transplant**, *n (%)* | <5 | 0 (0·0%) | 0 (0·0%) |
| **ICU admission**, *n (%)* | 348 (7·5%) | 1386 (9·7%) | 58 (33·0%) |
| Length of stay (hours), *median (IQR)*^#^ | 48·5 (30-87·3) | 34 (20-56·8) | 64 (36·8-144) |
| **Ventilation**, *n (%)* | 205 (4·4%) | 637 (4·4%) | 33 (18·8%) |
| Time spent on ventilation (hours), *median (IQR)*^#^ | 21 (13-42) | 21 (13-39) | 32 (18-107) |
| **Transfers** |  | n = 14341 |  |
| To a psychiatric ward/hospital, *n (%)* | 1840 (39·6%) | 5463 (38·1%) | 89 (50·6%) |
| To another hospital, *n (%)* | 303 (6·5%) | 2108 (14·7%) | N/A |
|  |  | n = 14341 |  |
| **In-hospital deaths**, *n (%)* | 14 (0·3%) | 63 (0·4%) | 7 (4·0%) |
|  | N = 3884 people | N = 11936 people | N= 174 people |
| **Total deaths by the end of the study period**, *n (%)* | 198 (5·1%) | 598 (5·0%) | 16 (9·2%) |

ICU = Intensive Care Unit; IQR = interquartile range; N/A = not applicable.

* Individuals in this group come from the non tox hospital group.

^Δ^ Does not include stay in a psychiatric unit.

^#^ Reported only for patients in an ICU/ventilated.

Supplementary Table 4**:** Model of care vs outcomes for accidental paracetamol poisoning-related admissions.

|  | **Tox Hospital** | **Non Tox Hospital** | **Transfer to Tox Hospital (including liver unit)*** |
| --- | --- | --- | --- |
| **Number of admissions** | 920 | 3171 | 87 |
| **Length of stay** (days), *median (IQR)*^Δ^ | 2 (2-5) | 2 (2-5) | 5 (3-12) |
| ≤1 day, *n (%)* | 17 (1·8%) | 65 (2·0%) | 0 (0·0%) |
| 2 days, *n (%)* | 516 (56·1%) | 1531 (48·3%) | <5 |
| >2 days, *n (%)* | 387 (42·1%) | 1575 (49·7%) | 83 (95·4%) |
| **Drug induced liver injury**, *n (%)* | 76 (8·3%) | 175 (5·5%) | 26 (29·9%) |
| **Dialysis**, *n (%)* | 16 (1·7%) | 41 (1·3%) | 6 (6·9%) |
| **Liver transplant**, *n (%)* | 0 (0·0%) | 0 (0·0%) | 0 (0·0%) |
| **ICU admission**, *n (%)* | 65 (7·1%) | 277 (8·7%) | 31 (35·6%) |
| Length of stay (hours), *median (IQR)*^#^ | 65 (40-204) | 40 (20-87) | 131 (48-317) |
| **Ventilation**, *n (%)* | 27 (2·9%) | 101 (3·2%) | 16 (18·4%) |
| Time spent on ventilation (hours), *median (IQR)*^#^ | 63 (21·5-142) | 40 (14-101) | 148·5 (40·3-256·5) |
| **Transfers** |  |  |  |
| To a psychiatric ward/hospital, *n (%)* | 55 (6·0%) | 218 (6·9%) | <5 |
| To another hospital, *n (%)* | 40 (4·3%) | 348 (11·0%) | N/A |
| **In-hospital deaths**, *n (%)* | 13 (1·4%) | 44 (1·4%) | 8 (9·2%) |
|  | N = 897 people | N = 3065 people | N = 86 people |
| **Total deaths by the end of the study period**, *n (%)* | 126 (14·0%) | 421 (13·7%) | 15 (17·4%) |

ICU = Intensive Care Unit; IQR = interquartile range; N/A = not applicable.

* Individuals in this group come from the non tox hospital group.

^Δ^ Does not include stay in a psychiatric unit.

^#^ Reported only for patients in an ICU/ventilated.

Supplementary Table 5**:** Model of care vs outcomes for undetermined/other paracetamol poisoning-related admissions.

|  | **Tox Hospital** | **Non Tox Hospital** | **Transfer to Tox Hospital (including liver unit)*** |
| --- | --- | --- | --- |
| **Number of admissions** | 338 | 676 | 16 |
| **Length of stay** (days), *median (IQR)*^Δ^ | 2 (2-3) | 2 (2-4) | 3 (2-14·5) |
| ≤1 day, *n (%)* | 9 (2·7%) | 38 (5·6%) | 0 (0·0%) |
| 2 days, *n (%)* | 216 (63·9%) | 368 (54·4%) | 5 (31·2%) |
| >2 days, *n (%)* | 113 (33·4%) | 270 (39·9%) | 11 (68·8%) |
| **Drug induced liver injury**, *n (%)* | 16 (4·7%) | 38 (5·6%) | 5 (31·2%) |
| **Dialysis**, *n (%)* | <5 | 14 (2·1%) | <5 |
| **Liver transplant**, *n (%)* | <5 | 0 (0·0%) | 0 (0·0%) |
| **ICU admission**, *n (%)* | 29 (8·6%) | 83 (12·3%) | 7 (43·8%) |
| Length of stay (hours), *median (IQR)*^#^ | 44 (24-110) | 42 (19·5-104·5) | 70 (18·5-152·5) |
| **Ventilation**, *n (%)* | 15 (4·4%) | 40 (5·9%) | 6 (37·5%) |
| Time spent on ventilation (hours), *median (IQR)*^#^ | 32 (13·5-35·5) | 49·5 (15·8-181·8) | 49 (27-62) |
| **Transfers** |  |  |  |
| To a psychiatric ward/hospital, *n (%)* | 43 (12·7%) | 97 (14·3%) | 7 (43·8%) |
| To another hospital, *n (%)* | 14 (4·1%) | 86 (12·7%) | N/A |
| **In-hospital deaths**, *n (%)* | <5 | 19 (2·8%) | <5 |
|  | N = 330 people | N = 657 people | N = 16 people |
| **Total deaths by the end of the study period**, *n (%)* | 34 (10·3%) | 84 (12·8%) | <5 |

ICU = Intensive Care Unit; IQR = interquartile range; N/A = not applicable.

* Individuals in this group come from the non tox hospital group.

^Δ^ Does not include stay in a psychiatric unit.

^#^ Reported only for patients in an ICU/ventilated.

Supplementary Table 6**:** Charlson groupings for people with an intentional paracetamol poisoning-related admission stratified by intentional poisoning repetition.

|  | **No Repeats** | **One Repeat Poisoning** | **More Than One Repeat Poisoning** | **Total** |
| --- | --- | --- | --- | --- |
| **Number of individuals** | 12098 | 2001 | 1373 | 15472 |
| **Charlson groupings**, *n (%)* |  |  |  |  |
| Myocardial infarction | 178 (1·5%) | 36 (1·8%) | 22 (1·6%) | 236 (1·5%) |
| Congestive heart failure | 154 (1·3%) | 30 (1·5%) | 28 (2·0%) | 212 (1·4%) |
| Peripheral vascular disease | 68 (0·6%) | 8 (0·4%) | 17 (1·2%) | 93 (0·6%) |
| Cerebrovascular disease | 210 (1·7%) | 45 (2·2%) | 42 (3·1%) | 297 (1·9%) |
| Dementia | 71 (0·6%) | 10 (0·5%) | 10 (0·7%) | 91 (0·6%) |
| Chronic pulmonary disease | 550 (4·5%) | 152 (7·6%) | 196 (14·3%) | 898 (5·8%) |
| Rheumatic disease | 48 (0·4%) | 10 (0·5%) | 11 (0·8%) | 69 (0·4%) |
| Peptic ulcer disease | 131 (1·1%) | 34 (1·7%) | 42 (3·1%) | 207 (1·3%) |
| Mild liver disease | 88 (0·7%) | 30 (1·5%) | 26 (1·9%) | 144 (0·9%) |
| Diabetes without chronic complication | 597 (4·9%) | 147 (7·3%) | 180 (13·1%) | 924 (6·0%) |
| Diabetes with chronic complication | 478 (4·0%) | 100 (5·0%) | 124 (9·0%) | 702 (4·5%) |
| Hemiplegia or paraplegia | 254 (2·1%) | 50 (2·5%) | 72 (5·2%) | 376 (2·4%) |
| Kidney disease | 328 (2·7%) | 80 (4·0%) | 80 (5·8%) | 488 (3·2%) |
| Any malignancy, including lymphoma and leukaemia, except malignant neoplasm of the skin | 536 (4·4%) | 82 (4·1%) | 56 (4·1%) | 674 (4·4%) |
| Moderate or severe liver disease | 258 (2·1%) | 75 (3·7%) | 72 (5·2%) | 405 (2·6%) |
| Metastatic solid tumour | 678 (5·6%) | 60 (3·0%) | 48 (3·5%) | 786 (5·1%) |
| AIDS/HIV | 84 (0·7%) | 24 (1·2%) | 36 (2·6%) | 144 (0·9%) |
| **Other comorbidities not captured by Charlson**, *n (%)* |  |  |  |  |
| Mental disorders due to known physiological conditions (F01-F09) | 540 (4·5%) | 158 (7·9%) | 239 (17·4%) | 937 (6·1%) |
| Mental and behavioural disorders due to psychoactive substance use (F10-F19) | 3877 (32·0%) | 1010 (50·5%) | 918 (66·9%) | 5805 (37·5%) |
| Schizophrenia, schizotypal, delusional, and other non-mood psychotic disorders (F20-F29) | 650 (5·4%) | 223 (11·1%) | 295 (21·5%) | 1168 (7·5%) |
| Mood [affective] disorders (F30-F39) | 5766 (47·7%) | 1485 (74·2%) | 1228 (89·4%) | 8479 (54·8%) |
| Anxiety, dissociative, stress-related, somatoform and other nonpsychotic mental disorders (F40-F48) | 6020 (49·8%) | 1480 (74·0%) | 1239 (90·2%) | 8739 (56·5%) |
| Behavioural syndromes associated with physiological disturbances and physical factors (F50-F59) | 505 (4·2%) | 212 (10·6%) | 308 (22·4%) | 1025 (6·6%) |
| Disorders of adult personality and behaviour (F60-F69) | 2413 (19·9%) | 917 (45·8%) | 1084 (79·0%) | 4414 (28·5%) |
| Intellectual disabilities (F70-F79) | 87 (0·7%) | 39 (1·9%) | 121 (8·8%) | 247 (1·6%) |
| Pervasive and specific developmental disorders (F80-F89) | 135 (1·1%) | 66 (3·3%) | 122 (8·9%) | 323 (2·1%) |
| Behavioural and emotional disorders with onset usually occurring in childhood and adolescence (F90-F98) | 480 (4·0%) | 201 (10·0%) | 264 (19·2%) | 945 (6·1%) |
| Unspecified mental disorder (F99) | 73 (0·6%) | 32 (1·6%) | 55 (4·0%) | 160 (1·0%) |

AIDS/HIV = acquired immunodeficiency syndrome/human immunodeficiency virus.

Supplementary Table 7A**:** Long-term mortality: Total deaths by the end of the study period and top 5 causes of death (including primary and underlying) for people who have had paracetamol and non-paracetamol poisoning-related admissions, *n (%)**.

| **Paracetamol Poisoning-Related Admissions** | | **Non-Paracetamol Poisoning-Related Admissions** | |
| --- | --- | --- | --- |
| **Number of individuals** | 19895 | **Number of individuals** | 75812 |
| **Total deaths by end of study period** | 1382 (6·9%) | **Total deaths by end of study period** | 10673 (14·1%) |
| **Deaths with known cause^#^** | N = 1001 | **Deaths with known cause^#^** | N = 7797 |
| **X70** Intentional self-harm by hanging, strangulation and suffocation | 169 (16·9%) | **I25.9** Chronic ischaemic heart disease, unspecified | 1066 (13·7%) |
| **X44** Accidental poisoning by and exposure to other and unspecified  drugs, medicaments and biological substances | 116 (11·6%) | **J44.9** Chronic obstructive pulmonary disease, unspecified | 836 (10·7%) |
| **T71** Asphyxiation | 91 (9·1%) | **I10** Essential (primary) hypertension | 788 (10·1%) |
| **I25.9** Chronic ischaemic heart disease, unspecified | 89 (8·9%) | **I50.0** Congestive heart failure | 663 (8·5%) |
| **T39.1** 4-Aminophenol derivatives | 84 (8·4%) | **I46.9** Cardiac arrest, unspecified | 642 (8·2%) |

* Groups are mutually exclusive, people with a paracetamol poisoning related admission have not been included in the alternate group.

^#^ Reported where cause of death data was available, until 31 December 2018.

Supplementary Table 7B**:** Long-term mortality: Total deaths by the end of the study period and top 5 causes of death (including primary and underlying) for people who have had paracetamol poisoning-related admissions, stratified by external cause, *n (%)**.

| **Intentional Poisoning** | | **Accidental Poisoning** | | **Undetermined Intent/Other** | |
| --- | --- | --- | --- | --- | --- |
| **Number of individuals** | 15472 | **Number of individuals** | 3944 | **Number of individuals** | 984 |
| **Total deaths by end of study period** | 776 (5·0%) | **Total deaths by end of study period** | 543 (13·8%) | **Total deaths by end of study period** | 117 (11·9%) |
| **Deaths with known cause^#^** | N = 560 | **Deaths with known cause^#^** | N = 392 | **Deaths with known cause^#^** | N = 91 |
| **X70** Intentional self-harm by hanging,  strangulation and suffocation | 149 (26·6%) | **I25.9** Chronic ischaemic heart disease,  unspecified | 46 (11·7%) | **X44** Accidental poisoning by and exposure to  other and unspecified drugs, medicaments and biological substances | 21 (23·1%) |
| **T71** Asphyxiation | 81 (14·5%) | **J44.9** Chronic obstructive pulmonary disease,  unspecified | 45 (11·5%) | **T39.1** 4-Aminophenol derivatives | 12 (13·2%) |
| **X44** Accidental poisoning by and exposure to  other and unspecified drugs, medicaments and biological substances | 76 (13·6%) | **A41.9** Sepsis, unspecified | 41 (10·5%) | **T40.2** Other opioids | 12 (13·2%) |
| **X64** Intentional self-poisoning by and exposure to other and unspecified drugs, medicaments and biological substances | 76 (13·6%) | **X44** Accidental poisoning by and exposure to  other and unspecified drugs, medicaments and biological substances | 37 (9·4%) | **X40** Accidental poisoning by and exposure to  nonopioid analgesics, antipyretics and  antirheumatics | 12 (13·2%) |
| **T42.4** Benzodiazepines | 66 (11·8%) | **J96.9** Respiratory failure, unspecified | 33 (8·4%) | **T42.4** Benzodiazepines | 11 (12·1%) |

* People may fit into more than one category due to having different poisoning events.

^#^ Reported where cause of death data was available, until 31 December 2018.

Supplementary Table 7C**:** Long term mortality: Total deaths by the end of the study period and top 5 causes of death (including primary and underlying) for people who have had intentional paracetamol poisoning-related admissions, stratified by repeat intentional poisonings, *n (%)*.

| **No Repeats** | | **One Repeat Poisoning** | | **More than One Repeat Poisoning** | |
| --- | --- | --- | --- | --- | --- |
| **Number of individuals** | 12098 | **Number of individuals** | 2001 | **Number of individuals** | 1373 |
| **Total deaths by end of study period** | 571 (4·7%) | **Total deaths by end of study period** | 101 (5·0%) | **Total deaths by end of study period** | 104 (7·6%) |
| **Deaths with known cause^#^** | N = 415 | **Deaths with known cause^#^** | N = 68 | **Deaths with known cause^#^** | N = 77 |
| **X70** Intentional self-harm by hanging,  strangulation and suffocation | 107 (25·8%) | **X70** Intentional self-harm by hanging,  strangulation and suffocation | 22 (32·4%) | **X44** Accidental poisoning by and exposure to  other and unspecified drugs, medicaments  and biological substances | 23 (29·9%) |
| **T71** Asphyxiation | 59 (14·2%) | **T42.4** Benzodiazepines | 12 (17·6%) | **T42.4** Benzodiazepines | 20 (26·0%) |
| **X64** Intentional self-poisoning by and  exposure to other and unspecified drugs,  medicaments and biological substances | 50 (12·0%) | **T71** Asphyxiation | 12 (17·6%) | **X70** Intentional self-harm by hanging,  strangulation and suffocation | 20 (26·0%) |
| **X44** Accidental poisoning by and exposure to  other and unspecified drugs, medicaments  and biological substances | 41 (9·9%) | **X44** Accidental poisoning by and exposure to  other and unspecified drugs, medicaments and  biological substances | 12 (17·6%) | **X64** Intentional self-poisoning by and  exposure to other and unspecified drugs,  medicaments and biological substances | 16 (20·8%) |
| **T39.1** 4-Aminophenol derivatives | 40 (9·6%) | **X64** Intentional self-poisoning by and  exposure to other and unspecified drugs,  medicaments and biological substances | 10 (14·7%) | **T40.2** Other opioids | 14 (18·2%) |

^#^ Reported where cause of death data was available, until 31 December 2018.

Supplementary Table 8**:** Hazard Ratios including 95% confidence intervals (CIs) for age and sex adjusted survival analysis by intent.

| **Intent type*** | **Hazard Ratio (95% CI)**^#^ |
| --- | --- |
| **Accidental vs Intentional** |  |
| Overall (unadjusted) | 2·97 (2·67, 3·30) |
| Overall (adjusted for age and sex) | 1·53 (1·36, 1·70) |
| **Accidental vs Intentional for age-sex subgroups** |  |
| For Females <40 years | 1·20 (0·78, 1·85) |
| For Males <40 years | 0·94 (0·63, 1·42) |
| For Females ≥40 years | 3·00 (2·57, 3·50) |
| For Males ≥40 years | 1·70 (1·41, 2·05) |

CI = confidence interval.

* Accidental and undetermined/other poisoning events have been combined due to low numbers.

^#^ After removing the undetermined/other group as sensitivity analysis, the overall adjusted Hazard Ratio was estimated at 1·49 (95% CI 1·32, 1·67).


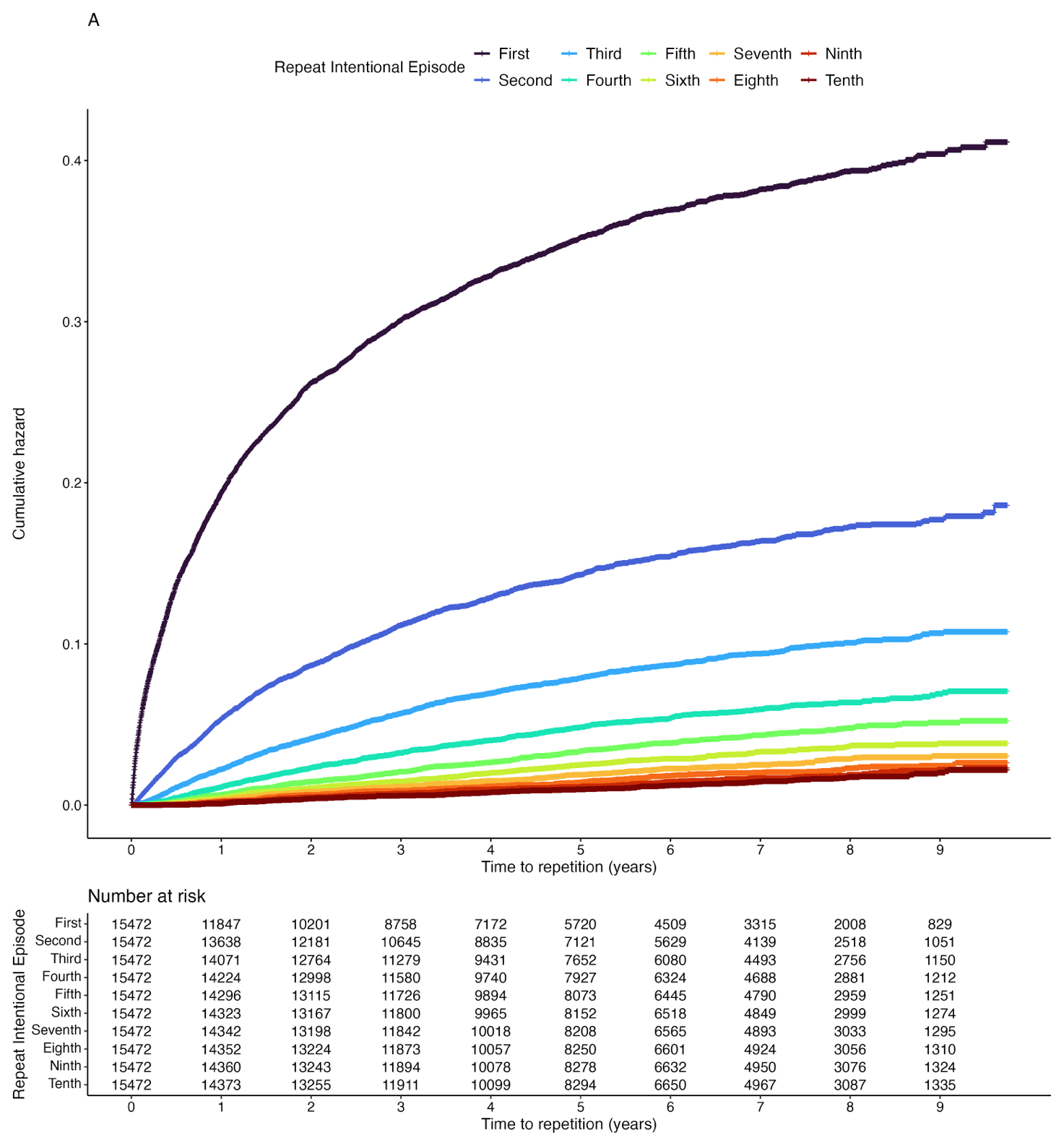


Supplementary Figure 1A**:** Time to intentional poisoning repetition over the entire study period as a cumulative incidence (N = 15,472).

**
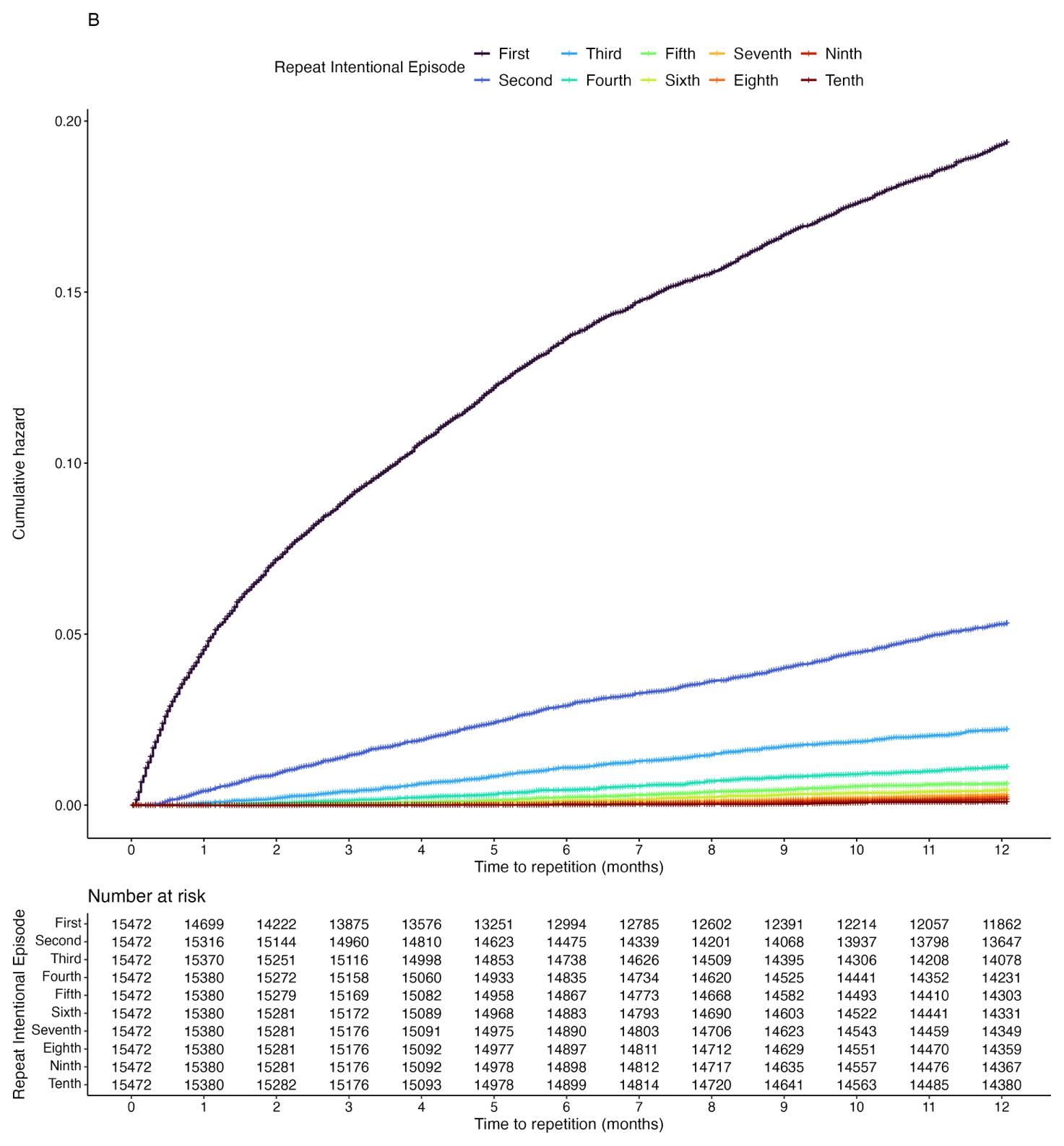
**

Supplementary Figure 1B**:** Time to intentional poisoning repetition over the first year as a cumulative incidence

(N = 15,472).

STROBE Statement—checklist of items that should be included in reports of observational studies^*^

|  | | **Item No** | **Recommendation** |  |
| --- | --- | --- | --- | --- |
| **Title and abstract** | | 1 | (*a*) Indicate the study’s design with a commonly used term in the title or the abstract  **Title** |  |
|  |  |  | (*b*) Provide in the abstract an informative and balanced summary of what was done and what was found  **Summary** |  |
| **Introduction** | | | |  |
| Background/rationale | | 2 | Explain the scientific background and rationale for the investigation being reported  **Research in context**  **Introduction** |  |
| Objectives | | 3 | State specific objectives, including any prespecified hypotheses  **Introduction** |  |
| **Methods** | | | |  |
| Study design | | 4 | Present key elements of study design early in the paper  **Methods, Design and setting** |  |
| Setting | | 5 | Describe the setting, locations, and relevant dates, including periods of recruitment, exposure, follow-up, and data collection  **Methods, Design and setting**  **Methods, Cohort selection and classification** |  |
| Participants | | 6 | (*a*) *Cohort study*—Give the eligibility criteria, and the sources and methods of selection of participants. Describe methods of follow-up  **Methods, Cohort selection and classification** |  |
|  |  |  | (*b*) *Cohort study*—For matched studies, give matching criteria and number of exposed and unexposed  **Not applicable** |  |
| Variables | | 7 | Clearly define all outcomes, exposures, predictors, potential confounders, and effect modifiers. Give diagnostic criteria, if applicable  **Methods, Cohort selection and classification**  **Supplementary Materials, Supplementary Methods** |  |
| Data sources/ measurement | | 8* | For each variable of interest, give sources of data and details of methods of assessment (measurement). Describe comparability of assessment methods if there is more than one group  **Methods, Cohort selection and classification**  **Methods, Statistical analysis**  **Supplementary Materials, Supplementary Methods** |  |
| Bias | | 9 | Describe any efforts to address potential sources of bias  **For our survival analysis we used the Cox Proportional Hazards model to adjust for age and sex. Other potential sources of bias are described in the Discussion/Limitations** |  |
| Study size | | 10 | Explain how the study size was arrived at  **Not applicable, this study covered the whole eligible population of NSW** |  |
| Quantitative variables | | 11 | Explain how quantitative variables were handled in the analyses. If applicable, describe which groupings were chosen and why  **Methods, Statistical analysis** |  |
| Statistical methods | | 12 | (*a*) Describe all statistical methods, including those used to control for confounding  **Methods, Statistical analysis** |  |
|  |  |  | (*b*) Describe any methods used to examine subgroups and interactions  **Methods, Statistical analysis** |  |
|  |  |  | (*c*) Explain how missing data were addressed  **Methods, Statistical analysis** |  |
|  |  |  | (*d*) *Cohort study*—If applicable, explain how loss to follow-up was addressed  **Potential loss to follow up from interstate/international migration is described in the Discussion, Limitations section. Loss to follow up from linkage errors is addressed in the Methods.** |  |
|  |  |  | (*e*) Describe any sensitivity analyses  **Methods, Statistical analysis** |  |
| **Results** | | | | |
| Participants | 13* | (a) Report numbers of individuals at each stage of study—eg numbers potentially eligible, examined for eligibility, confirmed eligible, included in the study, completing follow-up, and analysed  **Results**  **Results, Tables 1-3**  **Supplementary Materials, Supplementary Tables 1-7** | | |
|  |  | (b) Give reasons for non-participation at each stage  **Not applicable** | | |
|  |  | (c) Consider use of a flow diagram  **Not applicable** | | |
| Descriptive data | 14* | (a) Give characteristics of study participants (eg demographic, clinical, social) and information on exposures and potential confounders  **Results**  **Results, Tables 1-3**  **Supplementary Materials, Supplementary Tables 1-7** | | |
|  |  | (b) Indicate number of participants with missing data for each variable of interest  **Results, Tables 1-3**  **Supplementary Materials, Supplementary Tables 1-7** | | |
|  |  | (c) *Cohort study*—Summarise follow-up time (eg, average and total amount)  **Results, Demographics and outcomes of paracetamol poisonings**  **Results, Repeated intentional poisonings** | | |
| Outcome data | 15* | *Cohort study*—Report numbers of outcome events or summary measures over time  **Results**  **Results, Tables 1-3**  **Supplementary Materials, Supplementary Tables 1-7** | | |
| Main results | 16 | (*a*) Give unadjusted estimates and, if applicable, confounder-adjusted estimates and their precision (eg, 95% confidence interval). Make clear which confounders were adjusted for and why they were included  **Results, Long term outcomes and survival**  **Supplementary Materials, Supplementary Table 8** | | |
|  |  | (*b*) Report category boundaries when continuous variables were categorized  **Results, Tables 1-3**  **Supplementary Materials, Supplementary Tables 3-5, 8**  **Figures 1 and 2** | | |
|  |  | (*c*) If relevant, consider translating estimates of relative risk into absolute risk for a meaningful time period  **Not applicable** | | |
| Other analyses | 17 | Report other analyses done—eg analyses of subgroups and interactions, and sensitivity analyses  **Results**  **Results, Tables 1-3**  **Figures 3 and 4**  **Supplementary Materials, Supplementary Tables 1-8**  **Supplementary Materials, Supplementary Figure 1** | | |
| **Discussion** | | | | |
| Key results | 18 | Summarise key results with reference to study objectives  **Discussion** | | |
| Limitations | 19 | Discuss limitations of the study, taking into account sources of potential bias or imprecision. Discuss both direction and magnitude of any potential bias  **Discussion, Limitations** | | |
| Interpretation | 20 | Give a cautious overall interpretation of results considering objectives, limitations, multiplicity of analyses, results from similar studies, and other relevant evidence  **Discussion** | | |
| Generalisability | 21 | Discuss the generalisability (external validity) of the study results  **Discussion** | | |
| **Other information** | | | | |
| Funding | 22 | Give the source of funding and the role of the funders for the present study and, if applicable, for the original study on which the present article is based  **Declaration of interests statement** | | |

*Give information separately for cases and controls in case-control studies and, if applicable, for exposed and unexposed groups in cohort and cross-sectional studies.

**Note:** An Explanation and Elaboration article discusses each checklist item and gives methodological background and published examples of transparent reporting. The STROBE checklist is best used in conjunction with this article (freely available on the Web sites of PLoS Medicine at http://www.plosmedicine.org/, Annals of Internal Medicine at http://www.annals.org/, and Epidemiology at http://www.epidem.com/). Information on the STROBE Initiative is available at www.strobe-statement.org.
